# Cardioprotective medication adherence in Western Australians in the first year after myocardial infarction: restricted cubic spline analysis of adherence-outcome relationships

**DOI:** 10.1038/s41598-020-60799-5

**Published:** 2020-03-09

**Authors:** Melanie Greenland, Matthew W. Knuiman, Joseph Hung, Lee Nedkoff, Isabelle Arnet, Jamie M. Rankin, Monique F. Kilkenny, Frank M. Sanfilippo

**Affiliations:** 1 School of Population and Global Health, The University of Western Australia, Perth, Western Australia Australia; 2 Medical School, The University of Western Australia, Perth, Western Australia Australia; 3 Department of Pharmaceutical Sciences, Pharmaceutical Care Research Group, University of Basel, Basel, Switzerland; 4 Cardiology Department, Fiona Stanley Hospital, Murdoch, Western Australia Australia; 5 School of Clinical Sciences Monash Health, Monash University, Melbourne, Victoria Australia; 6 Stroke Division, Florey Institute of Neuroscience and Mental Health, University of Melbourne, Victoria, Australia

**Keywords:** Cardiology, Myocardial infarction, Drug therapy

## Abstract

Adherence to cardioprotective medications following myocardial infarction (MI) is commonly assessed using a binary threshold of 80%. We investigated the relationship between medication adherence as a continuous measure and outcomes in MI survivors using restricted cubic splines (RCS). We identified all patients aged ≥65 years hospitalised for MI from 2003–2008 who survived one-year post-discharge (n = 5938). Adherence to statins, beta-blockers, renin angiotensin system inhibitors (RASI) and clopidogrel was calculated using proportion of days covered to one-year post-discharge (landmark date). Outcomes were 1-year all-cause death and major adverse cardiac events (MACE) after the landmark date. Adherence-outcome associations were estimated from RCS Cox regression models. RCS analyses indicated decreasing risk for both outcomes above 60% adherence for statins, RASI and clopidogrel, with each 10% increase in adherence associated with a 13.9%, 12.1% and 18.0% decrease respectively in adjusted risk of all-cause death (all p < 0.02). Similar results were observed for MACE (all p < 0.03). Beta-blockers had no effect on outcomes at any level of adherence. In MI survivors, increasing adherence to statins, RASI, and clopidogrel, but not beta blockers, is associated with a decreasing risk of death/MACE with no adherence threshold beyond 60%. Medication adherence should be considered as a continuous measure in outcomes analyses.

## Introduction

Coronary heart disease (CHD) remains a leading cause of death worldwide despite effective evidence-based therapies^1^. Survivors of non-fatal CHD are also at high risk of recurrent vascular events^2,3^. The guidelines for secondary prevention after myocardial infarction (MI) recommend a multidrug regimen of a statin, beta-blocker, renin-angiotensin system inhibitor (RASI) and aspirin^4–6^. Dual antiplatelet therapy with aspirin and a P2Y_12_ platelet antagonist, such as clopidogrel, is also recommended for up to 12-months after ACS^4–6^. When these medications are taken in combination with smoking cessation, nearly 75% of recurrent vascular events may be prevented^7^. However, adherence to guideline-recommended secondary preventive medications is often suboptimal^8–11^, and non-adherence has been associated with increased risk of cardiovascular events and death^8,9,11–14^.

Most studies of medication adherence using administrative datasets have calculated proportion of days covered (PDC) for each medication^15,16^ and dichotomised adherence using PDC ≥80% as a ‘good’ adherence threshold^8,9,11–13^. However, this arbitrary threshold may not be the most appropriate for different medications or for predicting outcomes. Furthermore, using categorical thresholds does not allow for identification of a graded adherence-outcome association between a continuous exposure and outcomes. An alternative strategy for exploring the association without the assumption of linearity is to use restricted cubic splines (RCS)^17^. To our knowledge there are no previous studies using this method to examine the relationship between medication adherence after MI and cardiovascular outcomes. Thus, the aim of the study was to assess and measure the association between individual cardioprotective medications and subsequent one-year outcomes in a population-based cohort of MI survivors using RCS to assess the adherence-outcome relationships.

## Methods

### Data sources

The study protocol has been previously described^18^. Data for this study were obtained from two core data collections of the Western Australian Data Linkage System - the Hospital Morbidity Data Collection (HMDC) for hospital admissions and Mortality register^19^. The study dataset contained all hospital and death records for all patients who were hospitalised for CHD from 2003–2008. Both datasets were complete up to the end of 2010. We also had linked data from the Commonwealth Pharmaceutical Benefits Scheme (PBS)^20^ containing patient-level information on government subsidised drugs dispensed from mid-2002 to the end of 2009. The evidence-based drugs of interest were statins, beta-blockers, RASI and clopidogrel. In the PBS, patients pay a capped amount per drug (patient co-payment) and the government pays any remainder. If the patient paid for the medication in full, the record did not appear in the dataset. This affects cheap drugs such as aspirin, which can be purchased over-the-counter without prescription. Hence, aspirin was not considered in this study as the recording of aspirin in the PBS would be incomplete. Instead, we used clopidogrel as a surrogate marker of antiplatelet exposure in the cohort.

### Study cohort

Supplementary Fig. S1 shows the patient selection for the study cohort. The HMDC dataset identified residents of Western Australia (WA) aged ≥65 years who had their first hospitalisation between 2003–2008 with a principal discharge diagnosis of MI (International Classification of Diseases 10^th^ revision Australian Modification (ICD-10-AM) I21). Approximately 90–95% of Australians aged ≥65 years hold a concession card which entitles them to a low patient co-payment for the PBS medications^18^. Indigenous patients were excluded as other government subsidised medication programs existed for these patients^18^. Patients who died within one-year of the MI discharge date or had less than two supplies in this period for at least one of the drug groups of interest were also excluded.

### Identification of medications and dosing assumptions

Anatomical Therapeutic Chemical (ATC) codes were used to identify the relevant medication supplies in the dataset (Supplementary Table S1)^20^. Unlike aspirin, clopidogrel is captured in full in the dataset as it was the only P2Y_12_ platelet inhibitor that was PBS-authorised for secondary prevention during the study period. All beta-blockers were included except for oxprenolol and pindolol which are not used for secondary prevention. Since dosage information is not captured in the PBS dataset, we checked the registered product information for each drug and assumed that beta-blockers were used once per day except for metoprolol tartrate, carvedilol and propranolol which were two per day, and RASI drugs were assumed to have a dose of one per day except for captopril (three per day)^21^. All statins and clopidogrel were assumed to have a dose of one per day. The distribution of time from a supply date to the next supply date for each ATC code was then calculated for patients overall and the 75^th^ percentile of the distribution was used as the exposure duration^22^, after confirming that this was consistent with PBS prescriptions which are intended to approximate monthly supplies^23^.

### Adherence estimates

We adopted the landmark analysis method^24^ with landmark date being one-year after the MI discharge date to estimate the association between PDC adherence and adverse outcomes. PDC values were calculated for each drug group separately between the MI discharge date and landmark date^15,16^. The numerator was the number of days a patient was covered by the medication from the first supply date until landmark date. The denominator was the number of days from first supply date to the landmark date. If a patient obtained a new supply before their current supply ended, any overlap in days was counted once in the numerator. We defined users for each drug group as patients who had at least two supplies and therefore a non-zero PDC estimate for that drug group.

### Covariates

Baseline demographics, comorbidities and other relevant covariates were identified from the hospitalisation and PBS data. Residential postcode on MI admission was used to derive an Accessibility/Remoteness Index of Australia category which measures relative access to services (grouped as major cities, inner regional, outer regional, remote, very remote). Prior CHD in any diagnosis field and prior percutaneous or surgical coronary artery revascularisation procedure (CARP) were identified from hospitalisation data using a 15-year lookback from the MI admission date. We also identified readmissions for CHD including MI (as a principal discharge diagnosis) with or without a CARP within the one-year landmark period. Other comorbidities were similarly identified from hospitalisation data using a 15-year lookback from the one-year landmark date. Supplementary Table S2 shows diagnosis and procedure codes. Binary variables for concomitant drugs (yes/no) were derived for each of the four drug groups in the landmark period.

### Outcomes

Outcomes were identified in the one-year follow-up period after the landmark date. The primary outcome was all-cause death, and secondary outcome was a major adverse cardiac event (MACE), a composite of: all-cause death, ACS admission (ICD-10-AM I20.0 or I21 in the principal diagnosis field), stroke admission (ICD-10-AM I60, I61, I63, I64 in the principal diagnosis field), or admission for CARP in any procedure field, whichever event occurred first. Patients were censored at the end of the follow-up period if there were no specified outcomes.

### Statistical analysis

We compared patient characteristics for users of each drug group and for the total cohort. We fitted adjusted Cox regression models with RCSs to investigate the effect of adherence to each separate drug group on outcomes. All Cox models were adjusted for all baseline demographics, comorbidities, prior CARPs, and concomitant medications listed in Table 1. RCS Cox models determined the shape of the relationship between continuous PDC adherence and outcomes without any *a priori* assumption of linearity. RCSs fit a smooth continuous curve of adjusted HRs with 95% confidence intervals (CIs) across adherence levels, allowing for changes in the function at defined knot points (30%, 60%, 90%), and restricts the splines to linear relationships at the tail ends^17^. The knot points are arbitrary and do not force curvature or inflections at these points. The RCS plots were restricted to PDC ≥ 30% due to small frequencies below 30%. We chose a PDC of 95% as the reference value for the calculation of hazard ratios for adherence in the RCS Cox models because we wanted to compare against a high adherence value close to 100%. These plots were used to visually and statistically assess the nature of the relationship. If they showed a linear relationship between adherence and outcome across the range of PDC values, or above a turning point, then in further Cox regression models (without RCS), a continuous linear PDC adherence model was fitted for the entire PDC range (1–100%) or from turning point to 100%. Trend p-values were calculated in adjusted Cox regression models to assess the change in risk of events for a 10% increase in adherence. We also included interaction terms for sex*adherence and MI type*adherence in each model to determine if the effect of adherence on outcomes was different between males and females, and between the different types of MI (STEMI: ST elevation MI; NSTEMI: non-STEMI; and unspecified MI where the presence of ST elevation was not specified in the medical record). There were no statistically significant interactions for sex or MI type for any of the drug groups, and so the interaction terms were dropped from the models. All analyses were performed using SAS version 9.4 (Cary NC, USA).

**Table 1:** Patient demographics, characteristics and PDC adherence estimates by drug group and overall for users.

Characteristic	Statin user^a^ (N = 5179)	Beta-blocker user^a^ (N = 4598)	RASI user^a^ (N = 4896)	Clopidogrel user^a^ (N = 4198)	Total (N = 5938)
Male	3091 (59.7)	2665 (58.0)	2853 (58.3)	2525 (60.1)	3417 (57.5)
Age (years) mean (SD)	76.5 (6.9)	76.8 (7.1)	76.9 (7.2)	76.6 (7.0)	77.2 (7.3)
**MI type**					
STEMI	1722 (33.2)	1557 (33.9)	1637 (33.4)	1431 (34.1)	1877 (31.6)
NSTEMI	2970 (57.4)	2581 (56.1)	2746 (56.1)	2394 (57.0)	3429 (57.8)
Unspecified MI	487 (9.4)	460 (10.0)	513 (10.5)	373 (8.9)	632 (10.6)
**Accessibility/Remoteness**					
Major Cities	3020 (58.3)	2699 (58.7)	2867 (58.6)	2477 (59.0)	3476 (58.5)
Inner Regional	1595 (30.8)	1402 (30.5)	1504 (30.7)	1288 (30.7)	1803 (30.4)
Outer Regional	412 (8.0)	366 (8.0)	383 (7.8)	316 (7.5)	480 (8.1)
Remote	93 (1.8)	78 (1.7)	85 (1.7)	75 (1.8)	107 (1.8)
Very Remote	59 (1.1)	53 (1.2)	57 (1.2)	42 (1.0)	72 (1.2)
**Comorbidities**					
Hypertension	3909 (75.5)	3510 (76.3)	3721 (76.0)	3198 (76.2)	4505 (75.9)
Heart failure	1613 (31.1)	1528 (33.2)	1727 (35.3)	1264 (30.1)	2018 (34.0)
Atrial fibrillation	1524 (29.4)	1349 (29.3)	1510 (30.8)	1135 (27.0)	1843 (31.0)
Diabetes	1557 (30.1)	1391 (30.3)	1451 (29.6)	1263 (30.1)	1769 (29.8)
Chronic Obstructive Pulmonary Disease	877 (16.9)	654 (14.2)	864 (17.6)	728 (17.3)	1062 (17.9)
Stroke	458 (8.8)	418 (9.1)	448 (9.2)	389 (9.3)	567 (9.5)
Peripheral Vascular Disease	963 (18.6)	846 (18.4)	907 (18.5)	776 (18.5)	1113 (18.7)
Chronic Kidney Disease	1066 (20.6)	979 (21.3)	1027 (21.0)	864 (20.6)	1282 (21.6)
Prior coronary heart disease hospitalisation	2103 (40.6)	1886 (41.0)	1992 (40.7)	1769 (42.1)	2482 (41.8)
Prior coronary artery revascularisation procedures	802 (15.5)	657 (14.3)	703 (14.4)	710 (16.9)	882 (14.9)
**CHD hospitalisation during landmark period**^**b**^					
With coronary revascularisation	2676 (51.7)	2312 (50.3)	2428 (49.6)	2401 (57.2)	2813 (47.4)
No coronary revascularisation	554 (10.7)	536 (11.7)	526 (10.7)	462 (11.0)	650 (10.9)
**Concomitant drug groups**					
Statin user		4111 (89.4)	4329 (88.4)	3827 (91.2)	5179 (87.2)
Beta-blocker user	4111 (79.4)		3828 (78.2)	3365 (80.2)	4598 (77.4)
RASI user	4329 (83.6)	3828 (83.3)		3514 (83.7)	4896 (82.5)
Clopidogrel user	3827 (73.9)	3365 (73.2)	3514 (71.8)		4198 (70.7)
**PDC adherence estimate**					
Median (Q1, Q3)	86.8 (77.5, 92.1)	65.0 (49.5, 87.6)	87.6 (74.4, 93.4)	86.5 (72.8, 92.2)	
**PDC denominator (days)**					
Median (Q1, Q3)	360.0 (345.0, 363.0)	360.0 (340.0, 363.0)	359.0 (343.0, 363.0)	360.0 (347.0, 363.0)	
**Events in one-year follow-up**					
Major adverse cardiac event	792 (15.3)	774 (16.8)	829 (16.9)	692 (16.5)	1021 (17.2)
All-cause death	387 (7.5)	397 (8.6)	445 (9.1)	337 (8.0)	554 (9.3)

^a^Users can be in multiple drug groups.

^b^CHD admission during the landmark period with or without a coronary artery revascularisation procedure (CARP) during the index myocardial infarction admission or within the landmark period.

SD: standard deviation; MI: myocardial infarction; STEMI: ST elevation MI (ICD-10-AM codes I21.0-I21.3); NSTEMI: non-ST elevation MI (ICD-10-AM code I21.4); Unspecified MI (ICD-10-AM code I21.9); CHD, coronary heart disease; RASI, renin-angiotensin system inhibitor; PDC, proportion of days covered; Q1, lower quartile; Q3, upper quartile.

### Ethical approval

This study complies with the Declaration of Helsinki. Ethics approval and waivers of consent were obtained from human research ethics committees of the Departments of Health (Western Australian 2014/11 and Federal) and the University of Western Australia (RA/4/1/8065).

## Results

### Patient demographics, characteristics and adherence estimates

**Figure 1:**
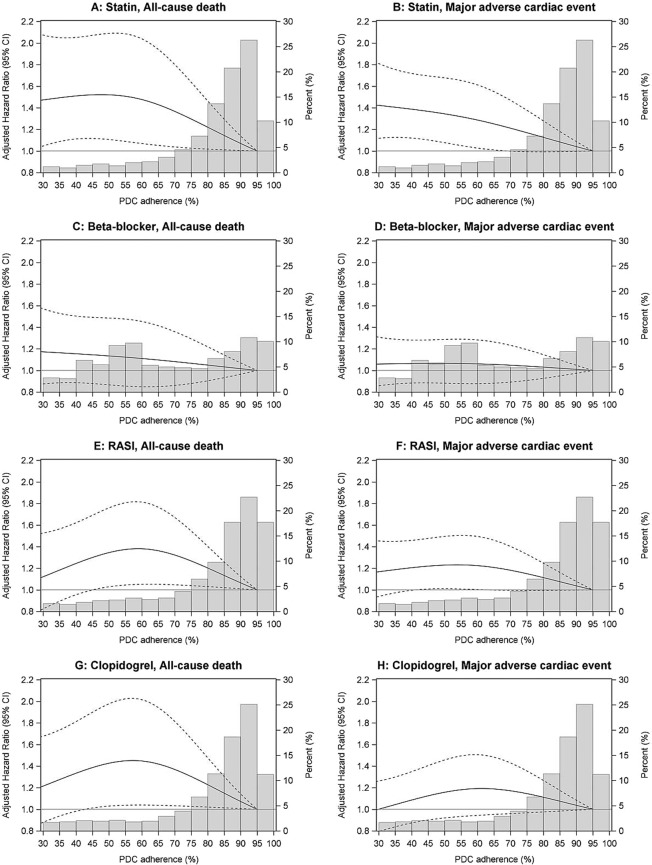
Fitted adjusted Cox regression restricted cubic spline models and proportion of days covered (PDC) histograms for users by drug group and outcome. The solid line is the adjusted hazard ratio compared to 95% PDC adherence as reference. Dashed lines are the upper and lower 95% confidence limits. The bars are the frequency distribution of adherence by 5% interval. The upper interval includes 100% adherence. Cox regression models with restricted cubic splines were adjusted for: age, sex, Accessibility/Remoteness Index of Australia, history of: hypertension, heart failure, atrial fibrillation, diabetes, chronic obstructive pulmonary disease, chronic kidney disease, stroke, peripheral vascular disease, coronary heart disease, coronary artery revascularisation procedure, coronary heart disease admissions with or without coronary artery revascularisation procedure in the one-year landmark period, and concomitant cardioprotective drugs.

There were 5938 patients in the study cohort. The majority were male (57.5%), the mean age was 77.2 (±7.3) years and the majority (58.5%) lived in major cities (Table 1). Most patients had two or more supplies for statins (87.2%) followed by RASI (82.5%), beta-blockers (77.4%) and clopidogrel (70.7%) during the landmark period. The age, sex distribution and clinical characteristics of patients were generally similar across the user drug groups (Table 1). However, a prior history of heart failure was more prevalent among RASI users while chronic obstructive pulmonary disease was less prevalent among beta-blocker users. Clopidogrel users were more likely to have a CARP during the landmark period. Users of each drug class were also likely to be users (>70%) of the other drug classes (Table 1). The median PDC adherence estimate for statins, beta-blockers, RASI and clopidogrel was 86.8%, 65.0%, 87.6% and 86.5% respectively, and the respective proportions with PDC ≥80% was 71.0%, 35.6%, 67.9%, and 66.3% (Fig. 1). Overall there were 554 (9.3%) all-cause deaths and 1021 (17.2%) MACE in the follow-up period.

The proportion of women who were identified as users of statins, RASI and clopidogrel was significantly lower than for men: 90 vs 83% (statins p < 0.0001), 83 vs 81% (RASI p = 0.01) and 74 vs 66% (clopidogrel p < 0.0001) (Supplementary Table S3). However, there was no significant difference in median adherence levels between women and men for these three drug groups, although there was a significantly lower median adherence in women observed for beta blockers in this univariate analysis (Supplementary Table S3).

### Restricted cubic spline models

Figure 1 shows the multivariable adjusted hazard ratios (HRs) and 95% confidence intervals (CIs) for the Cox regression models with RCSs for all-cause death and MACE for each drug group. The corresponding HRs and 95% CIs for various PDC values are shown in Supplementary Table S4. HRs for the other covariates in the models are shown in Supplementary Tables S5 and S6. For statins, there was a continuous reduction in risk of all-cause death above 60% adherence, and for MACE, across all PDC adherence values (Fig. 1A,B). For beta-blockers, there was no association between the PDC adherence estimate and risk of all-cause death or MACE (Fig. 1C,D). For RASI, increasing PDC adherence from 60% to 100% was associated with decreasing risk of all-cause death and MACE (Fig. 1E,F). Similar results were seen for clopidogrel (Fig. 1G,H).

### Adjusted hazard ratios according to PDC adherence levels

Table 2 shows the adjusted HRs for a 10% increase in PDC adherence for risk of all-cause death and MACE for each drug group. Among statin users, for each 10% increase in adherence between 1% and 100%, there was a 6.0% decrease in adjusted risk of all-cause death (p = 0.017) and a 5.4% decrease in adjusted risk of MACE (p = 0.003) (Table 2). When the analysis was restricted to statin users with PDC adherence ≥60%, the adjusted risk of all-cause death and MACE reduced by 13.9% and 10.4% respectively for every 10% increase in PDC adherence (both trend p < 0.02) (Table 2). There was no significant change in adjusted HRs for all-cause death or MACE for beta-blockers across the whole adherence range (Table 2).

**Table 2:** Adjusted hazard ratios for all-cause death and major adverse cardiac events by drug group for every 10% increase in medication adherence.

Drug group	N (%)	All-cause death		Major adverse cardiac event	
		Hazard ratio (95% CI)	Trend p-value	Hazard ratio (95% CI)	Trend p-value
**PDC 1–100%**					
Statin	5179 (100%)	0.940 (0.894, 0.989)	0.017	0.946 (0.912, 0.981)	0.003
Beta-blocker	4598 (100%)	0.976 (0.933, 1.020)	0.280	0.991 (0.959, 1.023)	0.561
**PDC 60–100%**					
Statin	4555 (88.0%)	0.861 (0.760, 0.976)	0.019	0.896 (0.820, 0.979)	0.015
Beta-blocker	2545 (55.4%)	0.934 (0.836, 1.045)	0.235	0.975 (0.900, 1.056)	0.531
RASI	4079 (83.3%)	0.879 (0.788, 0.979)	0.020	0.895 (0.825, 0.970)	0.007
Clopidogrel	3436 (81.8%)	0.820 (0.720, 0.934)	0.003	0.902 (0.822, 0.990)	0.029

CI, confidence interval; RASI, renin-angiotensin system inhibitor; PDC, proportion of days covered.

Cox regression models were adjusted for: age, sex, accessibility/remoteness, history of: hypertension, heart failure, atrial fibrillation, diabetes, chronic obstructive pulmonary disease, chronic kidney disease, stroke, peripheral vascular disease, coronary heart disease, coronary artery revascularisation procedure, coronary heart disease admissions with or without coronary artery revascularisation procedure in the one-year landmark period and concomitant cardioprotective drugs. Trend p-value is for a change in risk of outcomes for a 10% increase in adherence. Interaction terms for sex*adherence and MI type*adherence were not statistically significant for any of the drug groups (MI type is STEMI [ST elevation myocardial infarction], NSTEMI [non-STEMI] and unspecified myocardial infarction where presence of ST elevation was not specified in the medical record).

Because of the overall curvilinear relationships between adherence and outcomes for RASI and clopidogrel, but approximately linear relationships for PDC adherence ≥60%, we modelled PDC adherence for these two drugs for users with adherence between 60 and 100% only (Table 2). For RASI and clopidogrel, every 10% increase in PDC adherence was associated with a 12.1% and 18.0% decrease respectively in adjusted risk of all-cause death (both trend p < 0.02). The corresponding reductions for MACE were 10.5% and 9.8% respectively (both trend p < 0.03).

## Discussion

We evaluated the impact of adherence to guideline-recommended cardioprotective medications on fatal and non-fatal cardiovascular events in a population-based cohort of MI survivors aged ≥65 years. We used RCS analysis to demonstrate the adherence-outcome relationships for the individual drug groups. Firstly, we confirmed that adherence to evidence-based pharmacotherapies after recent MI was not ideal with only 35–71% of patients achieving a PDC adherence above the traditional value of 80% across the four drug groups. Secondly, using RCSs, we identified an approximately linear adherence-outcome relationship with outcomes for statins, curvilinear relationships for RASI and clopidogrel, and no significant association for beta-blockers. Thirdly, and most importantly, we showed that a binary adherence threshold of 80% for statins, RASI and clopidogrel does not provide an optimal measure of risk, with risk reduction below and above this adherence threshold. We also found that sex and MI type (STEMI, NSTEMI/unspecified MI) did not modify the effect of adherence on outcomes in adjusted regression analyses.

Our finding of generally suboptimal adherence to cardioprotective medications is concordant with other contemporary studies in ‘real-world’ coronary artery disease cohorts from similar high-income countries^8–11,14^. Medication adherence in MI survivors continues to decline with time and we have reported that for each incremental year since last ACS admission, there was an 8% increased odds of being dispensed fewer of the recommended drugs^10^. Previous evidence has suggested that secondary preventive medications together can mitigate risk for future events by nearly 75%^7^, but the benefits of these therapies are limited by adherence to treatment^25,26^. One way to improve adherence is with the use of polypills, with early trials showing promising results^27^. Individual studies and meta-analyses in MI and other coronary artery disease cohorts have confirmed that high adherence to statins, beta-blockers, RASI and platelet inhibitors, individually or together, are associated with a lower risk of death and major cardiovascular events^8,9,11–14^. However, these studies have generally dichotomised PDC ≥80% as indicating ‘good’ adherence and <80% as ‘poor’ or ‘non-adherence’ without testing if this threshold is optimal with respect to outcomes or the same for all drug groups^8,9,11–14^.

We therefore used RCSs to provide a visual and statistical assessment of the association between PDC adherence as a continuous exposure and outcomes for individual drug groups. The RCSs confirm an approximately linear association between adherence to statins and risk of MACE across the entire range of PDC values, while this relationship was apparent above 60% adherence for all-cause death. For RASI and clopidogrel, the RCSs appeared curvilinear with a turning point around 60% above which there were graded associations with all-cause death and MACE. These results suggest that significant reductions in major events can be achieved for adherence levels below 80% and that improvements in adherence levels above 80% for these three drug groups should be targeted because there is no plateauing of risk reduction.

In contrast, we found no significant association between PDC adherence to beta-blockers and cardiovascular outcomes, discordant with some previous studies^8,9^. However, it has been argued that beta-blocker therapy in the post-reperfusion era may provide little incremental survival benefit, and secondary prevention guidelines have questioned the benefit of indefinite beta-blocker therapy after an MI in people without significant left ventricular systolic dysfunction^4–6^. In support of this contention, one study found no association of beta-blocker use with cardiovascular events in stable patients with a prior history of MI^28^, and another study found that discontinuation of beta-blockers one-year after MI in patients without heart failure was not associated with higher subsequent mortality^29^. We showed a survival benefit with the other drug groups, but not beta-blockers, which suggests that it is not merely an epiphenomenon of “healthy adherer” effects. Furthermore, the shape of the RCSs for beta blockers are quite different to those of the other three drug groups. Nevertheless, our findings are retrospective and observational and warrant further investigation through prospective studies and clinical trials to confirm the place of beta blockers and their duration of use post-MI.

### Strengths and limitations

Our study used large linked data encompassing the entire population of WA and included complete outcomes. The study cohort was derived from population data and therefore enabled adherence to be estimated in a ‘real-world’ situation rather than strict clinical trial conditions. This makes the results more generalisable to the wider population. Although the study period was 2003–2010, the guidelines for secondary prevention pharmacotherapies after ACS^4,5^ have predominantly remained unchanged since this time implying that our findings are relevant to current practice. There were some limitations to the study. Adherence was calculated from claims data and records of a dispensing exist only if the pharmacy claimed for the cost. Dosing information is not recorded in the PBS database and we assumed each patient ingested and completed their supplied medications. We excluded Indigenous patients and non-concession card holders and therefore cannot generalise our results to these populations. We do not have data for other thienopyridine antiplatelet agents because clopidogrel was the only agent available on the PBS during the study period. We could not measure adherence to aspirin due to incomplete data for aspirin in the PBS dataset. We could not ascertain whether non-adherence was due to patient choice or if they were advised to discontinue for reasons such as drug side-effects. Despite adjustment for many potential confounders, we are unable to completely exclude a “healthy user” bias although our finding of a drug class-specific effect on survival suggests that this is not the case.

### Conclusions and implications

Understanding the association between adherence to guideline-recommended cardioprotective medications and cardiovascular outcomes is vital for preventing secondary events. Our findings reinforce the importance of optimal adherence especially to statins, RASI and antiplatelet drugs after a recent MI. Our study also suggests that use of RCSs is far more informative for interpreting relationships between medication adherence and outcomes than using the traditional 80% cut-off. An important clinical implication is that quality improvement efforts for patients with coronary artery disease must be expanded to include medication adherence as a key component of secondary prevention care. Our findings also highlight the importance of primary care in secondary prevention as general practitioners and pharmacists are now responsible for most of this activity, including prescription and monitoring of drug therapy after hospital discharge. Our findings reinforce the need for further research to reliably quantify effects of medication adherence and effects of specific combination of medications in populations of various risk and socioeconomic levels. The lack of benefit of beta blockers in MI survivors suggests that their role in long-term secondary prevention should be reassessed.

## Supplementary information

Supplementary information

## Data Availability

We will consider requests for data sharing on an individual basis, with the aim to share data whenever possible for appropriate research purposes. However, this research project uses data obtained from a third-party source under strict privacy and confidentiality agreements from Australian State and Federal government databases, which are governed by their ethics committees and data custodians. This was from the Western Australian Department of Health and the Australian Department of Health. The data were provided after approval was granted from their standard application processes for access to the linked datasets. Therefore, any requests to share these data with other researchers will be subject to formal approval from the third-party ethics committees and data custodian(s). Researchers interested in these data should contact the Client Services Team at the Data Linkage Branch of the Western Australian Department of Health (www.datalinkage-wa.org.au/contact-us).
