# Supplementary material for: Cardioprotective medication adherence in Western Australians in the first year after myocardial infarction: restricted cubic spline analysis of adherence-outcome relationships
Source: Sci Rep. 2020 Mar 9;10:4315. doi: 10.1038/s41598-020-60799-5 (PMC7062740; doi:10.1038/s41598-020-60799-5)
Supplement: Supplementary file 1 — Supplementary information [file 41598_2020_60799_MOESM1_ESM.pdf]

SUPPLEMENTARY FILE

Cardioprotective medication adherence in Western Australians in the first year after myocardial infarction: restricted cubic spline analysis of adherence-outcome relationships

Melanie Greenland, Matthew W Knuiman, Joseph Hung, Lee Nedkoff, Isabelle Arnet, Jamie M Rankin, Monique F Kilkenny, Frank M Sanfilippo.

Supplementary Figure S1: Flowchart of patient selection for the study cohort.

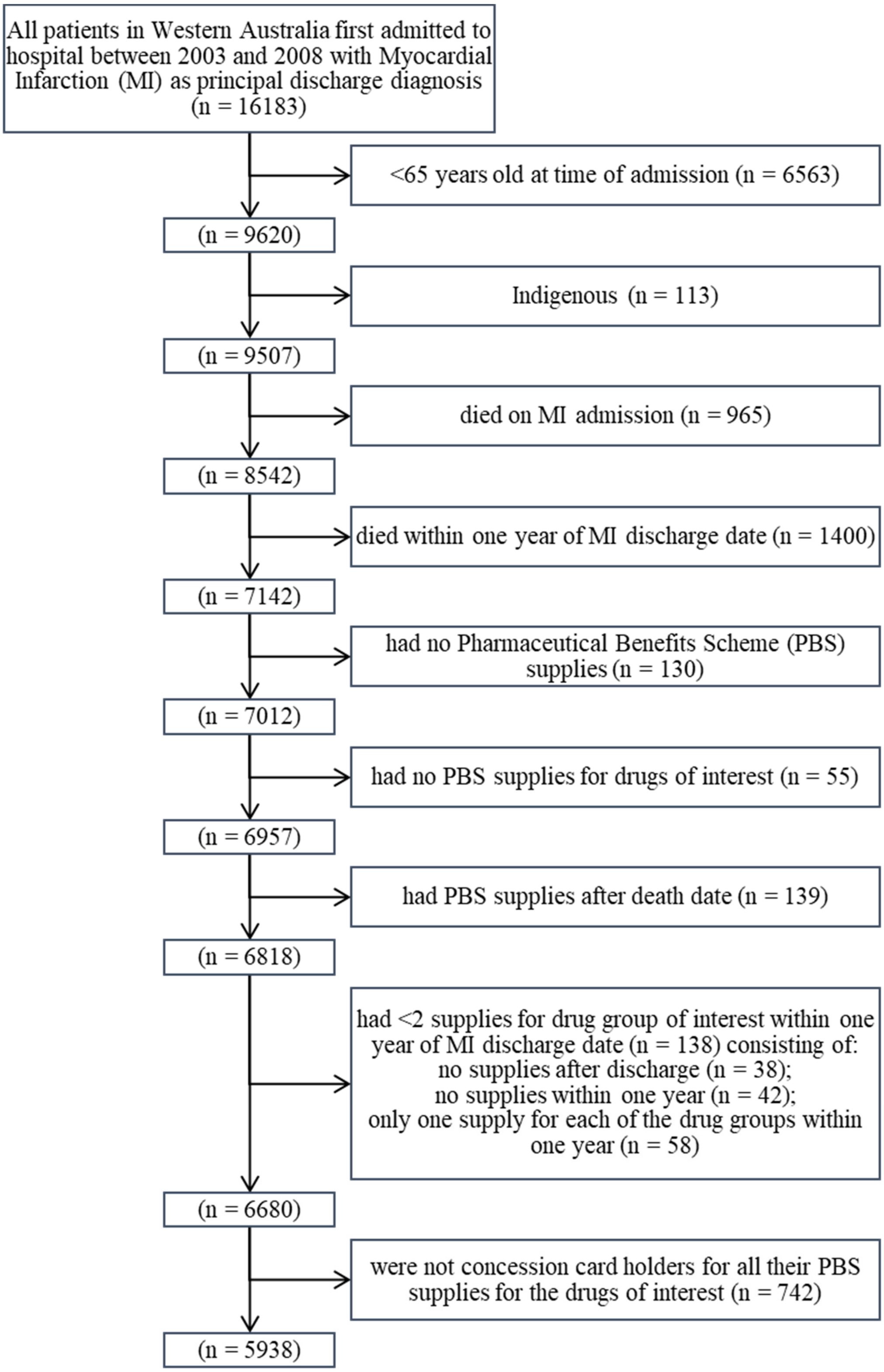

Supplementary Table S1: Anatomical Therapeutic Chemical (ATC) codes, dosing assumptions and frequency of supplies for medications in the study cohort.

ATC code	Category, Name	Dosing (per day)	Total supply frequency in study cohort
**B01A**	**Antithrombotic agents**		
B01AC04	Clopidogrel	1	45570
**C07**	**Beta-blocking agents**		
C07AA05	Propranolol (50)	1	1
C07AA05	Propranolol (100)	2	133
C07AA07	Sotalol	1	0
C07AB02	Metoprolol (tartrate)	2	15883
C07AB02	Metoprolol (succinate)	1	1092
C07AB03	Atenolol	1	8518
C07AB07	Bisoprolol	1	3015
C07AB11	s-Atenolol	1	0
C07AB12	Nebivolol	1	0
C07AG02	Carvedilol	2	3696
**C09**	**Agents acting on the renin-angiotensin system**		
**C09A**	**Ace inhibitors, plain**		
C09AA01	Captopril	3	150
C09AA02	Enalapril	1	1018
C09AA03	Lisinopril	1	781
C09AA04	Perindopril	1	9424
C09AA05	Ramipril	1	29209
C09AA06	Quinapril	1	662
C09AA09	Fosinopril	1	668
C09AA10	Trandolapril	1	738
**C09C**	**Angiotensin II antagonists, plain**		
C09CA01	Losartan	1	0
C09CA02	Eprosartan	1	212
C09CA03	Valsartan	1	2
C09CA04	Irbesartan	1	7181
C09CA06	Candesartan	1	2659
C09CA07	Telmisartan	1	1417
C09CA08	Olmesartan medoxomil	1	15
**C10**	**Lipid modifying agents**		
**C10AA**	**HMG-CoA reductase inhibitors (statins)**		
C10AA01	Simvastatin	1	14346
C10AA02	Lovastatin	1	0

ATC code	Category, Name	Dosing (per day)	Total supply frequency in study cohort
C10AA03	Pravastatin	1	4993
C10AA04	Fluvastatin	1	118
C10AA05	Atorvastatin	1	35115
C10AA07	Rosuvastatin	1	1671

Supplementary Table S2: Diagnosis and procedure codes used to derive covariates for regression models.

Comorbidity/Procedure	ICD codes (ICD-9-CM, ICD-10-AM)
Hypertension	401-405, I10-I15
Heart Failure	428, I50
Atrial Fibrillation	427.3, I48
Diabetes	250, E10-E14
Chronic Obstructive Pulmonary Disease	490-494, 496, J40-J47
Stroke	430, 431, 433, 434, 436, I60, I61, I63, I64
Peripheral Vascular Disease	440-448, I70-I79
Chronic Kidney Disease	*01Jan1988 to 30Jun1999*<br>250.4, 590.0, 590.2, 590.3, 590.8, 593.0, 593.1, 593.2, 593.6, 593.9, 599.7, 753.0, 753.1, 753.2, 753.3, 753.4, 996.1, V42.0, V45.1, 403, 404, 580-583, 585-589, V56, 405.01, 405.11, 405.91, 405.02, 405.12, 405.92, 593.81, 996.81, 996.73<br>*01Jul1999 to 31Dec2014*:<br>E10.2, E11.2, E12.2, E13.2, E14.2, I15.0, I15.1, N39.1, N39.2, T82.4, Z94.0, Z99.2, I12, I13, N00-N12, N14, N15, N16, N18, N19, N25-N28, Q60-Q63, Z49
Coronary Heart Disease	410-414, I20-I25
Coronary Artery Revascularisation Procedures	36.01, 36.02, 36.05, 36.06, 36.07, 36.10-36.19, 35335-00, 35341-00, 38309-00, 38315-00, 35304-00, 35305-00, 38303-00, 38300-00, 35338-00, 35338-01, 35344-00, 35344-01, 38312-00, 38312-01, 38318-00, 38318-01, 35310-00, 35310-01, 35310-02, 38306-00, 38306-01, 38306-02, 90218-00, 90218-01, 90218-02, 90218-03, 38497-00, 38497-01, 38497-02, 38497-03, 38497-04, 38497-05, 38497-06, 38497-07, 38500-01, 38500-02, 38500-03, 38500-04, 38500-05, 38503-00, 38503-01, 38503-02, 38503-03, 38503-04, 90201-00, 90201-01, 90201-02, 90201-03, 38500-00, 38503-05

ICD-9-CM = International Classification of Diseases, 9th revision, Clinical Modification (includes procedure codes); ICD-10-AM = International Classification of Diseases, 10th revision Australian Modification (includes procedure codes).

Supplementary Table S3: Comparison of usage and median adherence for the 4 drug groups between men and women in the study cohort.

**Drug group**	**Users, n (%)**			**Median PDC adherence (%)**		
	**Men (n=3417)**	**Women (n=2521)**	**p value**	**Men (n=3417)**	**Women (n=2521)**	**p value**
Statins	3091 (90.5)	2088 (82.8)	<0.0001	87.0	86.7	0.13
Beta blockers	2665 (78.0)	1933 (76.7)	0.23	66.2	63.0	0.03
RASI	2853 (83.5)	2043 (81.0)	0.01	87.8	87.2	0.07
Clopidogrel	2525 (73.9)	1673 (66.4)	<0.0001	86.7	86.2	0.18

PDC: proportion of days covered; RASI: renin-angiotensin system inhibitors; p value for usage comparison is the chi-squared test; p value for adherence comparison is the Wilcoxon rank-sum test with continuity correction.

Supplementary Table S4: Adjusted hazard ratios (HR) and 95% confidence intervals (CI) for various PDC adherence levels compared to 95% as reference, by drug group and outcome.

Drug group	PDC adherence level	All-cause death Adjusted HR (95% CI)	MACE Adjusted HR (95% CI)
Statin	50	1.52 (1.11, 2.09)	1.34 (1.08, 1.68)
	60	1.48 (1.07, 2.04)	1.29 (1.03, 1.61)
	70	1.37 (1.04, 1.80)	1.21 (1.00, 1.47)
	80	1.22 (1.02, 1.46)	1.13 (0.99, 1.28)
	90	1.07 (1.01, 1.14)	1.04 (1.00, 1.09)
	95	1.00	1.00
Beta-blocker	50	1.13 (0.87, 1.49)	1.07 (0.88, 1.29)
	60	1.11 (0.85, 1.45)	1.06 (0.88, 1.28)
	70	1.08 (0.86, 1.36)	1.05 (0.89, 1.23)
	80	1.05 (0.90, 1.22)	1.03 (0.93, 1.15)
	90	1.02 (0.97, 1.07)	1.01 (0.97, 1.05)
	95	1.00	1.00
RASI	50	1.35 (1.03, 1.76)	1.23 (1.01, 1.50)
	60	1.38 (1.05, 1.81)	1.22 (1.00, 1.49)
	70	1.32 (1.05, 1.67)	1.18 (1.00, 1.40)
	80	1.20 (1.03, 1.40)	1.11 (0.99, 1.25)
	90	1.06 (1.01, 1.12)	1.04 (1.00, 1.08)
	95	1.00	1.00
Clopidogrel	50	1.43 (1.03, 1.98)	1.16 (0.92, 1.46)
	60	1.45 (1.04, 2.02)	1.19 (0.94, 1.51)
	70	1.37 (1.03, 1.81)	1.17 (0.96, 1.43)
	80	1.23 (1.02, 1.48)	1.11 (0.98, 1.27)
	90	1.07 (1.01, 1.14)	1.04 (0.99, 1.08)
	95	1.00	1.00

PDC, proportion of days covered; RASI, renin-angiotensin system inhibitor; MACE, major adverse cardiac event.

Supplementary Table S5: Adjusted hazard ratios (HR) and 95% confidence intervals (CI) for covariates in Cox regression models for all-cause death by drug group.

Covariate	All PDC values		PDC limited to 60-100%			
	Statin user* (n=5179)	Beta-blocker* user (n=4598)	Statin user*† (n=4555)	Beta-blocker user*† (n=2545)	RASI user*† (n=4079)	Clopidogrel user*† (n=3436)
Female	0.75 (0.61, 0.93)	0.79 (0.64, 0.97)	0.78 (0.62, 0.99)	0.85 (0.65, 1.13)	0.82 (0.66, 1.02)	0.78 (0.60, 1.00)
Age (years)	1.06 (1.05, 1.08)	1.06 (1.04, 1.07)	1.06 (1.04, 1.08)	1.06 (1.04, 1.08)	1.07 (1.06, 1.09)	1.06 (1.05, 1.09)
Accessibility/Remoteness Index of Australia						
Major Cities	1.00	1.00	1.00	1.00	1.00	1.00
Inner Regional	1.06 (0.85, 1.33)	1.03 (0.82, 1.29)	1.06 (0.83, 1.36)	1.10 (0.82, 1.49)	1.07 (0.85, 1.34)	1.33 (1.02, 1.72)
Outer Regional	0.99 (0.67, 1.47)	0.94 (0.64, 1.38)	1.06 (0.70, 1.59)	1.09 (0.67, 1.78)	0.77 (0.49, 1.19)	0.88 (0.52, 1.48)
Remote	0.62 (0.23, 1.67)	0.66 (0.25, 1.79)	0.55 (0.17, 1.72)	0.52 (0.13, 2.13)	0.99 (0.41, 2.42)	0.64 (0.20, 2.02)
Very Remote	1.88 (0.88, 4.01)	1.57 (0.64, 3.84)	1.20 (0.38, 3.79)	0.95 (0.23, 3.89)	1.08 (0.40, 2.91)	2.25 (0.83, 6.14)
Comorbidities						
Hypertension	0.97 (0.72, 1.30)	0.96 (0.72, 1.27)	0.90 (0.66, 1.24)	0.74 (0.50, 1.11)	0.84 (0.63, 1.12)	0.83 (0.59, 1.16)
Heart failure	1.66 (1.32, 2.09)	1.81 (1.44, 2.28)	1.63 (1.27, 2.09)	1.69 (1.25, 2.29)	1.62 (1.27, 2.06)	1.66 (1.26, 2.19)
Atrial fibrillation	1.51 (1.22, 1.86)	1.34 (1.09, 1.65)	1.41 (1.12, 1.76)	1.47 (1.12, 1.94)	1.34 (1.08, 1.67)	1.66 (1.29, 2.12)
Diabetes	1.25 (1.01, 1.55)	1.19 (0.96, 1.48)	1.18 (0.93, 1.49)	1.06 (0.80, 1.41)	1.29 (1.03, 1.62)	0.98 (0.75, 1.28)
Chronic Obstructive Pulmonary Disease	1.41 (1.12, 1.77)	1.45 (1.15, 1.82)	1.47 (1.14, 1.88)	1.49 (1.09, 2.02)	1.69 (1.34, 2.13)	1.53 (1.17, 2.00)
Chronic Kidney Disease	1.42 (1.13, 1.77)	1.56 (1.25, 1.94)	1.37 (1.07, 1.75)	1.64 (1.22, 2.21)	1.39 (1.10, 1.75)	1.50 (1.14, 1.96)
Stroke	1.55 (1.19, 2.00)	1.62 (1.26, 2.08)	1.60 (1.21, 2.12)	1.69 (1.22, 2.35)	1.79 (1.38, 2.32)	1.57 (1.17, 2.12)
Peripheral Vascular Disease	1.47 (1.18, 1.84)	1.29 (1.03, 1.61)	1.47 (1.15, 1.87)	1.45 (1.08, 1.95)	1.30 (1.02, 1.65)	1.51 (1.15, 1.97)

Covariate	All PDC values		PDC limited to 60-100%			
	Statin user* (n=5179)	Beta-blocker* user (n=4598)	Statin user*† (n=4555)	Beta-blocker user*† (n=2545)	RASI user*† (n=4079)	Clopidogrel user*† (n=3436)
CHD history						
Prior CHD	1.23 (0.97, 1.55)	1.33 (1.06, 1.67)	1.20 (0.93, 1.55)	1.38 (1.01, 1.88)	0.96 (0.76, 1.22)	1.11 (0.83, 1.48)
Prior CARP	0.84 (0.63, 1.12)	0.75 (0.56, 1.01)	0.93 (0.68, 1.26)	0.76 (0.52, 1.11)	0.92 (0.66, 1.27)	0.94 (0.67, 1.31)
CHD admission during one-year landmark period						
With CARP‡	0.50 (0.39, 0.65)	0.50 (0.38, 0.66)	0.45 (0.34, 0.61)	0.52 (0.36, 0.76)	0.49 (0.36, 0.66)	0.59 (0.44, 0.80)
Without CARP‡	1.05 (0.80, 1.38)	1.17 (0.91, 1.51)	1.04 (0.78, 1.40)	1.24 (0.89, 1.73)	1.08 (0.82, 1.42)	1.23 (0.90, 1.67)
Neither	1.00	1.00	1.00	1.00	1.00	1.00
Concomitant drugs						
Statin user	-	0.58 (0.45, 0.74)	-	0.59 (0.42, 0.83)	0.64 (0.50, 0.83)	0.71 (0.51, 0.98)
Beta-blocker user	1.00 (0.79, 1.28)	-	0.91 (0.70, 1.17)	-	1.17 (0.92, 1.50)	1.12 (0.84, 1.51)
RASI user	1.09 (0.84, 1.43)	1.01 (0.78, 1.30)	1.10 (0.82, 1.48)	0.99 (0.69, 1.42)	-	1.06 (0.77, 1.45)
Clopidogrel user	1.07 (0.86, 1.34)	1.05 (0.84, 1.30)	1.01 (0.79, 1.28)	1.06 (0.79, 1.42)	0.99 (0.79, 1.24)	-

*Users can be in multiple drug groups.
†Only includes users with PDC 60-100%.
‡CHD admission during the landmark period with or without a CARP during the index myocardial infarction admission or within the landmark period.
CHD, coronary heart disease; CARP, coronary artery revascularisation procedure; RASI, renin-angiotensin system inhibitor.

Supplementary Table S6: Adjusted hazard ratios (HR) and 95% confidence intervals (CI) for covariates in Cox regression models for MACE (major adverse cardiac event) outcome by drug group.

**Covariate**	**All PDC values**		**PDC limited to 60-100%**			
	**Statin user* (n=5179)**	**Beta-blocker* user (n=4598)**	**Statin user*† (n=4555)**	**Beta-blocker user*† (n=2545)**	**RASI user*† (n=4079)**	**Clopidogrel user*† (n=3436)**
Female	0.88 (0.76, 1.02)	0.87 (0.75, 1.01)	0.90 (0.76, 1.05)	0.87 (0.75, 1.01)	0.84 (0.72, 0.99)	0.84 (0.70, 1.00)
Age (years)	1.04 (1.03, 1.06)	1.04 (1.03, 1.05)	1.04 (1.03, 1.06)	1.04 (1.03, 1.05)	1.05 (1.04, 1.06)	1.04 (1.03, 1.06)
Accessibility/Remoteness Index of Australia						
Major Cities	1.00	1.00	1.00	1.00	1.00	1.00
Inner Regional	0.97 (0.83, 1.13)	0.98 (0.83, 1.15)	0.94 (0.79, 1.12)	0.98 (0.83, 1.15)	0.95 (0.81, 1.13)	1.01 (0.84, 1.21)
Outer Regional	0.89 (0.67, 1.19)	0.84 (0.63, 1.13)	0.93 (0.69, 1.26)	0.84 (0.63, 1.13)	0.71 (0.51, 0.98)	0.76 (0.53, 1.09)
Remote	0.80 (0.42, 1.49)	0.88 (0.47, 1.64)	0.73 (0.36, 1.48)	0.88 (0.47, 1.64)	0.83 (0.43, 1.61)	0.77 (0.38, 1.56)
Very Remote	1.38 (0.76, 2.52)	1.10 (0.54, 2.22)	1.05 (0.47, 2.36)	1.10 (0.54, 2.22)	0.94 (0.45, 2.00)	0.92 (0.38, 2.24)
Comorbidities						
Hypertension	1.14 (0.93, 1.41)	1.05 (0.86, 1.29)	1.10 (0.88, 1.39)	1.05 (0.86, 1.29)	0.98 (0.79, 1.21)	1.10 (0.86, 1.40)
Heart failure	1.37 (1.17, 1.61)	1.48 (1.26, 1.74)	1.32 (1.11, 1.57)	1.48 (1.26, 1.74)	1.29 (1.09, 1.53)	1.34 (1.11, 1.62)
Atrial fibrillation	1.18 (1.02, 1.37)	1.12 (0.96, 1.30)	1.16 (0.99, 1.36)	1.12 (0.96, 1.30)	1.14 (0.97, 1.34)	1.28 (1.07, 1.53)
Diabetes	1.33 (1.15, 1.55)	1.34 (1.15, 1.56)	1.36 (1.16, 1.60)	1.34 (1.15, 1.56)	1.45 (1.23, 1.71)	1.18 (0.99, 1.41)
Chronic Obstructive Pulmonary Disease	1.26 (1.07, 1.49)	1.26 (1.06, 1.49)	1.31 (1.10, 1.57)	1.26 (1.06, 1.49)	1.47 (1.23, 1.75)	1.35 (1.11, 1.64)
Chronic Kidney Disease	1.32 (1.12, 1.55)	1.47 (1.25, 1.73)	1.32 (1.11, 1.58)	1.47 (1.25, 1.73)	1.30 (1.09, 1.55)	1.34 (1.10, 1.62)
Stroke	1.31 (1.08, 1.59)	1.36 (1.12, 1.65)	1.33 (1.07, 1.64)	1.36 (1.12, 1.65)	1.42 (1.16, 1.75)	1.36 (1.09, 1.70)
Peripheral Vascular Disease	1.26 (1.08, 1.49)	1.13 (0.96, 1.34)	1.26 (1.06, 1.51)	1.13 (0.96, 1.34)	1.11 (0.92, 1.33)	1.35 (1.12, 1.64)

Covariate	All PDC values		PDC limited to 60-100%			
	**Statin user* (n=5179)**	**Beta-blocker* user (n=4598)**	**Statin user*† (n=4555)**	**Beta-blocker user*† (n=2545)**	**RASI user*† (n=4079)**	**Clopidogrel user*† (n=3436)**
CHD history						
Prior CHD	1.51 (1.28, 1.79)	1.46 (1.24, 1.73)	1.51 (1.26, 1.82)	1.46 (1.24, 1.73)	1.19 (1.00, 1.42)	1.49 (1.22, 1.82)
Prior CARP	1.15 (0.96, 1.39)	1.11 (0.91, 1.35)	1.17 (0.96, 1.44)	1.11 (0.91, 1.35)	1.23 (0.99, 1.53)	1.12 (0.90, 1.40)
CHD admission during one-year landmark period						
With CARP‡	0.74 (0.62, 0.88)	0.73 (0.61, 0.88)	0.70 (0.58, 0.84)	0.73 (0.61, 0.88)	0.75 (0.61, 0.91)	0.92 (0.75, 1.12)
Without CARP‡	1.35 (1.11, 1.64)	1.43 (1.18, 1.73)	1.37 (1.11, 1.69)	1.43 (1.18, 1.73)	1.40 (1.13, 1.72)	1.47 (1.16, 1.85)
Neither	1.00	1.00	1.00	1.00	1.00	1.00
Concomitant drugs						
Statin user	-	0.70 (0.57, 0.85)	-	0.70 (0.57, 0.85)	0.65 (0.53, 0.80)	0.71 (0.55, 0.90)
Beta-blocker user	1.17 (0.98, 1.40)	-	1.07 (0.88, 1.29)	-	1.15 (0.95, 1.38)	1.20 (0.97, 1.49)
RASI user	1.02 (0.85, 1.23)	0.97 (0.81, 1.16)	1.05 (0.85, 1.29)	0.97 (0.81, 1.16)	-	1.00 (0.80, 1.24)
Clopidogrel user	1.17 (0.99, 1.37)	1.18 (1.00, 1.39)	1.23 (1.02, 1.47)	1.18 (1.00, 1.39)	1.05 (0.89, 1.26)	-

*Users can be in multiple drug groups.
†Only includes users with PDC 60-100%.
‡CHD admission during the landmark period with or without a CARP during the index myocardial infarction admission or within the landmark period.
CHD, coronary heart disease; CARP, coronary artery revascularisation procedure; RASI, renin-angiotensin system inhibitor.
